# Cervical Osteomyelitis With Paraspinal Abscess Caused by Salmonella Typhi in an Immunocompetent Adult: A Case Report

**DOI:** 10.7759/cureus.87510

**Published:** 2025-07-08

**Authors:** Aqeel Saleem, Abla Agha, Zaid Al Hassani, Abdulfatah Hanoun, Ali Al Hassani

**Affiliations:** 1 Department of Infectious Diseases, Sheikh Tahnoon Medical City, Al Ain, ARE; 2 College of Medicine, University of Sharjah, Sharjah, ARE; 3 Department of Internal Medicine, Sheikh Tahnoon Medical City, Al Ain, ARE; 4 Department of Internal Medicine, Tawam Hospital, Al Ain, ARE

**Keywords:** cervical spine infection, extraintestinal salmonellosis, immunocompetent host, paraspinal abscess, salmonella typhi, vertebral osteomyelitis

## Abstract

*Salmonella *Typhi infection typically presents as gastrointestinal illness but may rarely lead to severe systemic complications. Osteomyelitis is a recognized, although uncommon, extraintestinal manifestation that most often occurs in immunocompromised individuals or those with hemoglobinopathies. We report a rare case of cervical vertebral osteomyelitis and paraspinal abscess caused by *S. *Typhi in a previously healthy 37-year-old male. Initial imaging with CT and MRI revealed deep cervical and paraspinal abscesses with inflammatory changes suggestive of osteomyelitis, but no spinal canal involvement. The diagnosis was established through culture of intraoperative abscess fluid. Despite initial clinical improvement, recurrence of the abscess necessitated three surgical debridements and vacuum-assisted closure therapy. The patient completed an eight-week course of intravenous ertapenem and oral azithromycin and achieved full clinical and radiological resolution. This case underscores the importance of considering *Salmonella* species in the differential diagnosis of vertebral infections, even in immunocompetent hosts.

## Introduction

Salmonellosis is a globally prevalent infection with clinical manifestations ranging from self-limiting gastroenteritis to serious systemic complications such as bacteremia, meningitis, and osteomyelitis [1]. Although *Staphylococcus aureus *and *Staphylococcus epidermidis *are the most common causative organisms of osteomyelitis, *Salmonella *species are recognized as rare etiologic agents. *Salmonella *osteomyelitis accounts for approximately 0.8% of all reported *Salmonella *infections and about 0.45% of all osteomyelitis cases [2,3]. The most common route of bone involvement is hematogenous spread during episodes of bacteremia [1,4-7].

*Salmonella *osteomyelitis can be classified into typhoidal (caused by *Salmonella enterica *serovars Typhi and Paratyphi) and non-typhoidal forms. Typhoidal vertebral osteomyelitis is exceptionally rare and has been described in only a small number of case reports [8]. In a review by Santos and Sapico, 54% of *Salmonella *osteomyelitis cases occurred in patients with predisposing conditions such as sickle cell disease, atherosclerosis, diabetes mellitus, collagen vascular disorders, liver cirrhosis, or achlorhydria, whereas 46% occurred in individuals without identifiable risk factors [9]. Although vertebral osteomyelitis is more frequently observed in immunocompromised patients, it can occasionally present in otherwise healthy adults [10,11].

The most common clinical features of vertebral osteomyelitis are localized spinal pain and fever; however, these symptoms are nonspecific and can delay diagnosis [10,11]. We report a rare case of *Salmonella *Typhi cervical vertebral osteomyelitis with associated paraspinal abscess in an immunocompetent adult. This report is significant as it highlights the diagnostic challenges of spinal infections in patients without classic risk factors and emphasizes the need to consider *Salmonella* species in the differential diagnosis, particularly in individuals with recent travel to endemic areas or a remote history of enteric fever.

## Case presentation

A 37-year-old Pakistani male with no known comorbidities presented to the emergency department in the United Arab Emirates with a two-week history of fever, posterior neck pain, progressive neck swelling, occipital headache, and dizziness. He denied recent infections, trauma, or other constitutional symptoms. He also denied smoking, alcohol use, or illicit substance use. The swelling was localized to the posterior cervical region, measuring approximately 10 cm in diameter, erythematous, fluctuant, and tender, with yellowish purulent discharge.

The patient had last traveled internationally nine months prior to presentation, during which he visited his home country, Pakistan, and developed an episode of infectious diarrhea. Based on verbal history, he was diagnosed with enteric fever via stool culture and treated with a combination of intravenous ceftriaxone followed by oral azithromycin for a total of 14 days. Symptoms resolved completely. Given the current delayed focal presentation and absence of clear new exposure, a chronic carrier state was suspected.

On examination, the patient was afebrile, hemodynamically stable, and neurologically intact. Initial investigations, including complete blood count, renal and liver function panels, HbA1c, HIV screening, serum immunoglobulins (IgG, IgA, IgM, and IgE), and complement levels (C3 and C4), were within normal limits.

**Figure 1:**
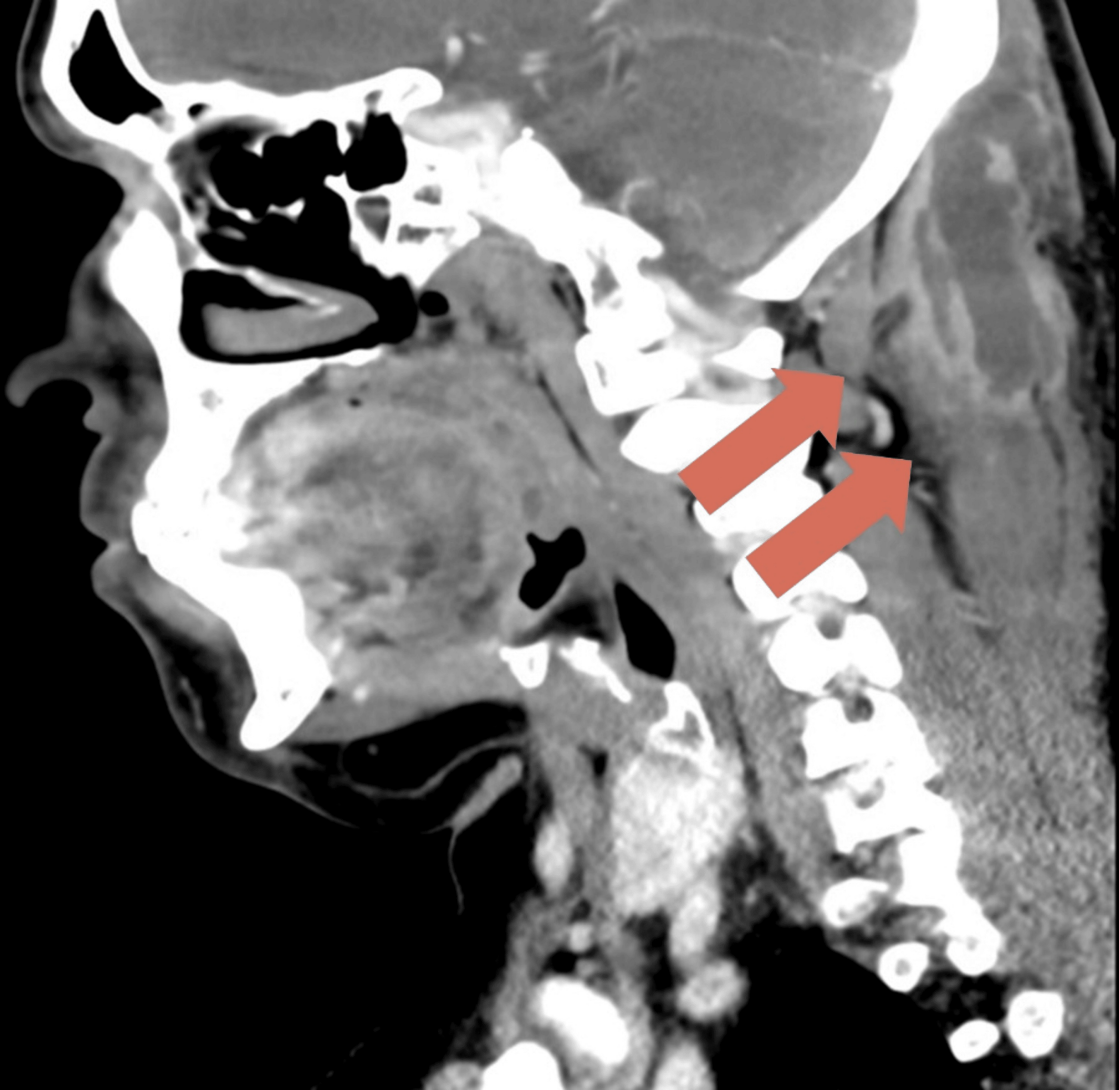
Contrast-enhanced CT of the cervical spine demonstrating deep paraspinal abscess Contrast-enhanced CT scan of the cervical spine on hospital day 1 showing a rim-enhancing fluid collection measuring 6.6 × 3.5 × 1.6 cm. The abscess extends from the occipital region to the level of the C2 vertebra. No bony destruction or intracranial extension is identified.

**Figure 2:**
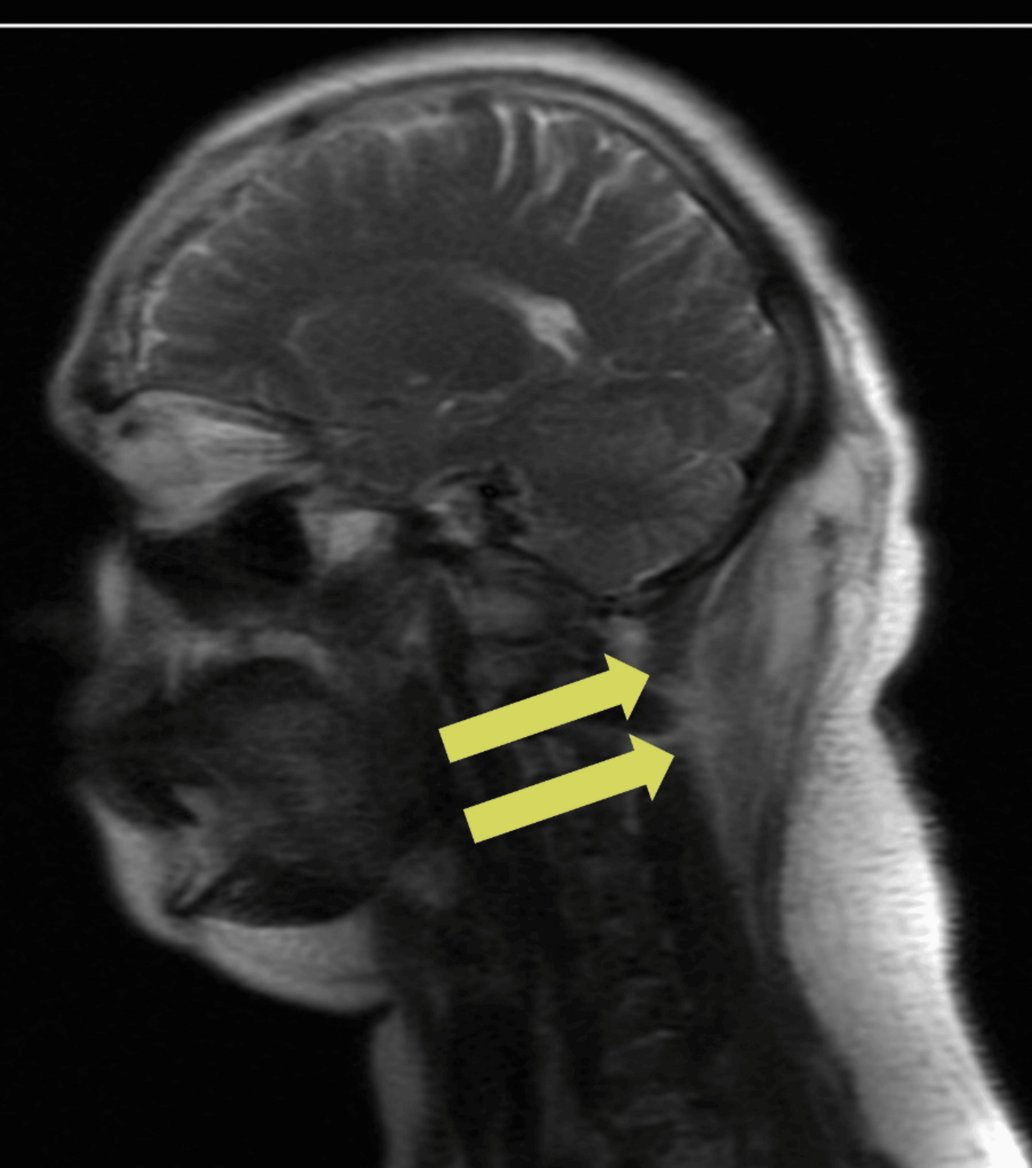
MRI showing abscess within the semispinalis capitis muscle and bone changes suggestive of osteomyelitis Axial T1-weighted post-contrast MRI of the upper cervical spine performed on hospital day 1 shows a 4 × 2 × 6 cm abscess within the right semispinalis capitis muscle. Surrounding inflammatory changes are present, suggestive of underlying osteomyelitis. There is no involvement of the spinal canal or intracranial structures.

On the day of admission (hospital day 1), a contrast-enhanced CT scan of the cervical spine was performed. Imaging revealed a rim-enhancing abscess measuring 6.6 × 3.5 × 1.6 cm, extending from the occipital region to the level of the C2 vertebra, with no evidence of bony erosion or intracranial extension (Figure 1). MRI of the brain and upper cervical spine identified a second abscess within the right semispinalis capitis muscle, measuring 4 × 2 × 6 cm. Surrounding inflammatory changes were noted, suggestive of underlying osteomyelitis. There was no involvement of the spinal canal or intracranial structures (Figure 2).

On hospital day 2, the patient underwent urgent surgical debridement with drainage of the abscess and placement of a surgical drain, performed by the neurosurgery team. Examination immediately following the procedure confirmed successful evacuation of the abscess cavity. Empiric intravenous antibiotic therapy was initiated with amoxicillin-clavulanate and clindamycin. By hospital day 4, the surgical drain was removed due to a marked reduction in output. The patient demonstrated rapid clinical improvement, with complete resolution of fever within 24 hours of surgery.

Intraoperative cultures grew *S*. Typhi. Culture results became available by hospital day 5. Blood cultures obtained prior to the initiation of antibiotics remained negative. Based on antimicrobial susceptibility testing (Table 1), antibiotic therapy was escalated to intravenous meropenem and azithromycin.

**Table 1:** Antibiotic susceptibility profile of Salmonella Typhi isolated from cervical abscess Antibiotic susceptibility profile of the *Salmonella enterica* serovar Typhi isolate obtained from intraoperative abscess fluid culture. The organism demonstrated resistance to amoxicillin-clavulanate and fluoroquinolones and sensitivity to meropenem and azithromycin. Susceptibility testing was completed by hospital day 5 and guided the adjustment of antibiotic therapy. MIC, minimum inhibitory concentration; R, resistant; S, sensitive

Drug	MIC interpretation	MIC dilution
Amoxicillin/clavulanate	S	
Ampicillin	R	
Cefotaxime	R	
Ceftazidime	R	
Ciprofloxacin	R	
Meropenem	S	
Piperacillin/tazobactam	S	≤4
Tetracycline	S	
Trimethoprim/sulfa	R	

Despite initial clinical improvement, the patient developed persistent wound drainage by hospital day 14. A repeat contrast-enhanced CT scan revealed reaccumulation of the abscess, now enlarged to 5.8 × 4.5 × 3.3 cm. A second surgical debridement was performed on hospital day 15, followed by the initiation of vacuum-assisted closure (VAC) therapy. Due to incomplete wound resolution, a third surgical debridement was required on hospital day 21. Intraoperative cultures were repeated during the second and third debridements; however, no additional organisms were isolated.

Evaluation for alternative causes of infection or immunosuppression (including complete blood count, HIV screening, serum immunoglobulin panel, complement levels, and HbA1c) was unremarkable. The VAC device was removed after one week, on hospital day 28, with continued improvement in wound healing.

He initially received intravenous meropenem in combination with azithromycin starting on hospital day 5, based on culture sensitivity results. On hospital day 14, meropenem was switched to ertapenem to facilitate discharge, while azithromycin was continued.

**Figure 3:**
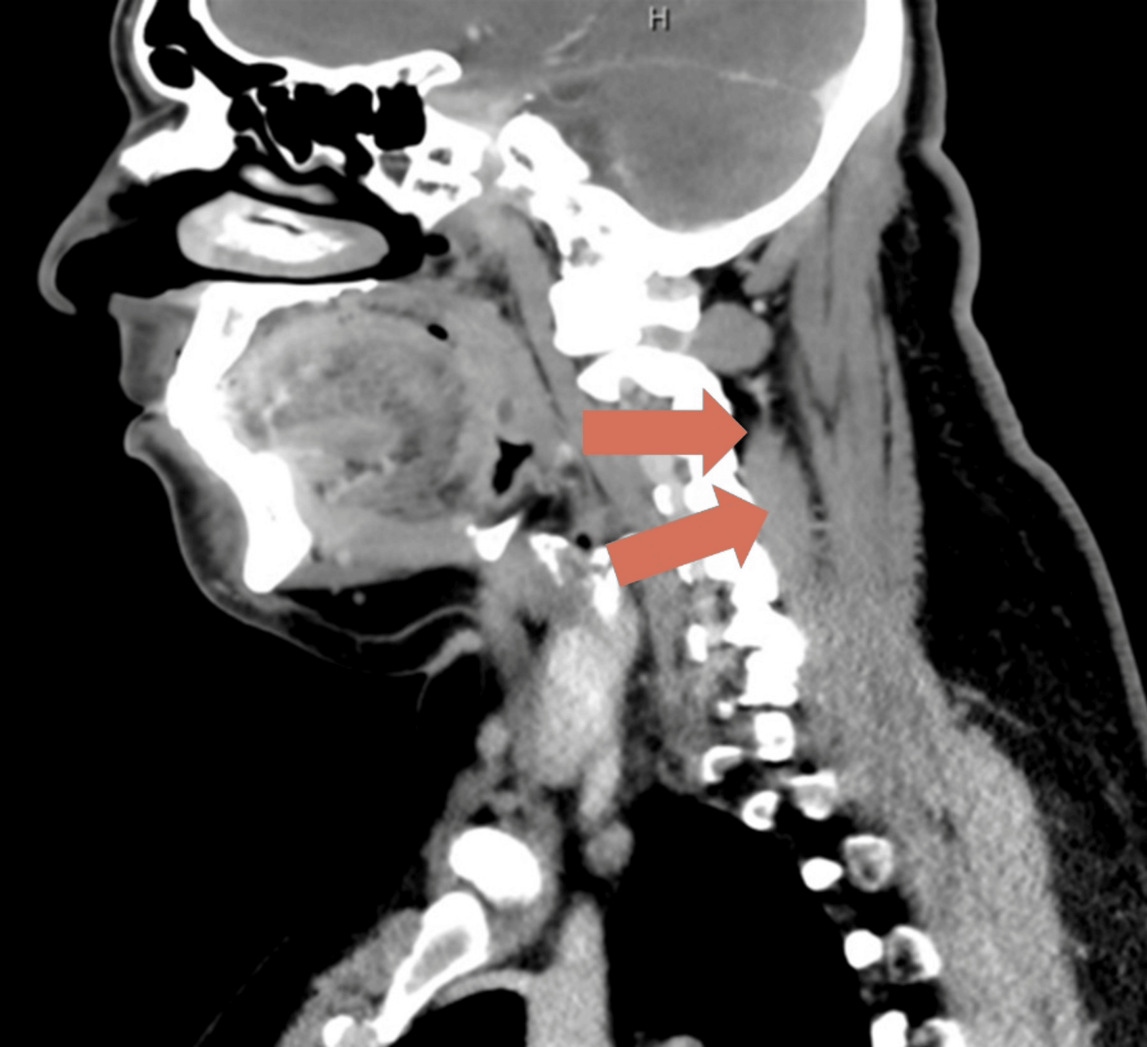
Complete resolution of cervical abscess on follow-up imaging Contrast-enhanced CT of the cervical spine obtained during outpatient follow-up showed complete resolution of the previously documented posterior cervical abscess. No residual fluid collections or soft tissue inflammation are seen.

The patient was discharged on hospital day 30 to continue outpatient parenteral antibiotic therapy with once-daily intravenous ertapenem and oral azithromycin, completing a total of eight weeks of antibiotics from the time of the last surgical drainage. Follow-up imaging confirmed complete resolution of the cervical and paraspinal abscesses (Figure 3). The surgical wound healed without complications, inflammatory markers normalized, and the patient remained asymptomatic during outpatient follow-up.

Other potential causes of cervical osteomyelitis, including *Mycobacterium tuberculosis*, fungal infections, and other opportunistic pathogens, were considered and ruled out based on negative cultures, unremarkable immunologic workup, and absence of systemic features.

## Discussion

*Salmonella* has long been recognized as a rare but established cause of osteomyelitis, with reports dating back over a century [1]. Osteomyelitis due to *Salmonella *can be broadly divided into two categories: typhoid osteomyelitis, caused by *Salmonella *Typhi or *Salmonella *Paratyphi, and non-typhoid *Salmonella *osteomyelitis [2]. While bone infections due to *Salmonella* remain rare in general, involvement of the vertebral column, particularly the cervical spine, is extremely uncommon and has only been reported sporadically [2,5-7]. In our case, the patient was immunocompetent and lacked traditional risk factors such as hemoglobinopathies or chronic illnesses [9]. However, he reported an episode of enteric fever nine months prior following travel to South Asia, which resolved after a course of oral antibiotics. It is plausible that transient bacteremia during that infection allowed hematogenous seeding of the cervical vertebrae, leading to a delayed manifestation of osteomyelitis. Similar delayed presentations of *Salmonella *vertebral infection have been documented in the literature [12,13].

In a systematic review by Huang et al., vertebral involvement occurred in approximately 36 percent of adult cases of *Salmonella* osteomyelitis, with the lumbar spine being most frequently affected, followed by the thoracic spine. Cervical vertebrae were involved in only a minority of cases [2]. Our patient, who presented with isolated cervical vertebral osteomyelitis, fits a rare clinical profile that has been underrepresented in the literature. Reports by Reddy et al. and Toofan et al. describe similar presentations, although those cases involved younger or immunocompromised patients [5,7].

Although our patient had no identifiable comorbidities or immunodeficiency, he had a history of recent travel to South Asia and an episode of presumed enteric fever nine months prior. This could represent a transient bacteremia that allowed hematogenous seeding of the cervical vertebrae. Such latent presentations have been described even in immunocompetent individuals, suggesting that host immunity alone may not always prevent localized metastatic complications from systemic *Salmonella *infection [9,12,13].

The likely pathogenesis in this case involves hematogenous spread from a prior enteric infection, with possible persistence in reticuloendothelial reservoirs leading to delayed seeding of cervical tissue. Although typically seen in immunocompromised individuals, chronic carriage in an otherwise healthy host may permit rare metastatic infections. Surgical management, including repeated debridement and VAC therapy, was essential given the complexity and recurrence, while antibiotic therapy was guided by susceptibility and tailored for outpatient feasibility.

*Salmonella *osteomyelitis is commonly associated with hemoglobinopathies, most notably sickle cell disease, as well as other comorbidities such as diabetes, liver cirrhosis, malignancy, and immunosuppressive therapy [2,3,9-14]. However, a substantial proportion of cases occur in immunocompetent individuals without any predisposing factors. Santos and Sapico reported that nearly half of their cases involved patients without any identifiable risk factor [9]. Our case supports this observation, emphasizing that *Salmonella *can cause deep-seated infections even in otherwise healthy adults with no apparent immunodeficiency or recent trauma.

The clinical presentation of *Salmonella* vertebral osteomyelitis is typically nonspecific. Localized spinal pain is the most common symptom, but constitutional signs such as fever may be absent in up to one-third of patients [2,9]. This was evident in our case, where the patient presented solely with neck pain and no systemic symptoms, contributing to a delay in diagnosis. Given these diagnostic challenges, a high index of suspicion is essential, and early imaging should be considered in patients with unexplained vertebral pain, regardless of febrile status. Notably, our patient was entirely afebrile at presentation, further reinforcing the need for early imaging in patients with localized spinal symptoms even in the absence of systemic signs. In skull and spinal osteomyelitis, clinical features may include focal neurologic deficits, headache, seizures, or swelling, particularly when abscess formation is present [15].

Although rare, cases of delayed-onset osteomyelitis following typhoid fever have been reported, with latency periods ranging from several months to decades. Hurt et al. and Herbert and Ruskin described patients who developed vertebral osteomyelitis more than 30 years after initial infection, raising the possibility of chronic carrier states or bacterial persistence in bone tissue [12,13]. In our case, the patient had a history of presumed enteric fever nine months prior to the onset of symptoms. Although he experienced full clinical recovery at the time, it is plausible that transient bacteremia led to subclinical hematogenous seeding of the cervical spine. This supports the notion that even immunocompetent individuals may develop delayed focal infections following typhoidal illness and that early resolution of enteric fever does not necessarily preclude future localized complications.

Antibiotic therapy remains the cornerstone of treatment. Third-generation cephalosporins and fluoroquinolones are most frequently employed, often for a duration of six to eight weeks [9,16,17]. Azithromycin was continued alongside carbapenem therapy due to its intracellular penetration, efficacy against *S. *Typhi, and synergistic role in targeting persistent or biofilm-associated bacteria in osteomyelitis. This dual approach was intended to optimize pathogen clearance given the deep-seated infection and initial poor response. However, increasing resistance to cephalosporins and fluoroquinolones has been documented in various regions, including South Asia, the Middle East, and sub-Saharan Africa [16]. Lamichhane et al. highlighted growing concerns over multidrug-resistant *Salmonella *strains and underscored the importance of antimicrobial stewardship and molecular surveillance [16].

In the present case, initial empiric therapy with amoxicillin-clavulanate and clindamycin was ineffective. Culture and sensitivity testing of the abscess fluid revealed resistance to multiple commonly used agents, including ampicillin, cefotaxime, ceftazidime, ciprofloxacin, and trimethoprim-sulfamethoxazole. The isolate was sensitive to meropenem and azithromycin, prompting escalation to this combination on hospital day 5. Meropenem was later transitioned to once-daily ertapenem upon discharge for outpatient parenteral therapy, while azithromycin was continued throughout. This approach was guided by local resistance patterns, culture results, and the need for prolonged, biofilm-penetrating coverage against *Salmonella *in osteomyelitis.

Although our patient did not exhibit spinal instability or focal neurologic deficits, his course was complicated by neurological symptoms such as occipital headache and dizziness, as well as recurrent paraspinal abscesses. These complications necessitated prolonged hospitalization and three surgical interventions. Spinal abscesses caused by *Salmonella*, including paraspinal and epidural types, may require urgent surgical intervention when associated with mass effect, neurological symptoms, or failure of antibiotic therapy [18]. Surgical management is reserved for patients with extensive bony destruction, abscesses, or spinal instability. Procedures such as laminectomy, anterior debridement, or vertebral fusion may be required in complicated cases [9,18].

In our patient, initial surgical drainage was followed by persistent wound drainage and imaging-confirmed abscess reaccumulation. These complications required two additional debridements. VAC therapy was initiated after the second surgery and ultimately contributed to the resolution of the infection and successful wound healing.

## Conclusions

*Salmonella*-induced spinal infections are rare manifestations, typically reported in isolated case studies and more often observed in immunocompromised or elderly individuals. These pathogens, while generally causing gastrointestinal illness, can occasionally lead to serious metastatic complications such as vertebral osteomyelitis. The growing prevalence of antimicrobial-resistant *Salmonella *strains has made treatment increasingly challenging. In this case, a previously healthy adult developed cervical vertebral osteomyelitis with a paraspinal abscess nine months after a presumed enteric fever episode. Persistent infection, antibiotic resistance, and abscess recurrence required three surgical debridements and prolonged intravenous therapy. This case underscores the need to consider *Salmonella *in the differential diagnosis of spinal infections, even in immunocompetent hosts with resolved typhoidal illness and no apparent comorbidities.
